# A Sensitive Sensor Cell Line for the Detection of Oxidative Stress Responses in Cultured Human Keratinocytes

**DOI:** 10.3390/s140711293

**Published:** 2014-06-25

**Authors:** Ute Hofmann, Melanie Priem, Christine Bartzsch, Thomas Winckler, Karl-Heinz Feller

**Affiliations:** 1 Department of Medical Engineering and Biotechnology, University of Applied Sciences Jena, Carl-Zeiss-Promenade 2, 07745 Jena, Germany; E-Mails: ute.hofmann@fh-jena.de (U.H.); melanie.priem@fh-jena.de (M.P.); christine.bartzsch@fh-jena.de (C.B.); 2 Department of Pharmaceutical Biology, Institute of Pharmacy, Friedrich-Schiller-University of Jena, Semmelweisstraβe 10, 07743 Jena, Germany; E-Mail: t.winckler@uni-jena.de

**Keywords:** cell-based assay, whole-cell biosensor, heat shock protein, reporter gene, oxidative stress

## Abstract

In the progress of allergic and irritant contact dermatitis, chemicals that cause the generation of reactive oxygen species trigger a heat shock response in keratinocytes. In this study, an optical sensor cell line based on cultured human keratinocytes (HaCaT cells) expressing green fluorescent protein (GFP) under the control of the stress-inducible HSP70B' promoter were constructed. Exposure of HaCaT sensor cells to 25 μM cadmium, a model substance for oxidative stress induction, provoked a 1.7-fold increase in total glutathione and a ∼300-fold induction of transcript level of the gene coding for heat shock protein HSP70B'. An extract of *Arnica montana* flowers resulted in a strong induction of the HSP70B' gene and a pronounced decrease of total glutathione in keratinocytes. The HSP70B' promoter-based sensor cells conveniently detected cadmium-induced stress using GFP fluorescence as read-out with a limit of detection of 6 μM cadmium. In addition the sensor cells responded to exposure of cells to *A. montana* extract with induction of GFP fluorescence. Thus, the HaCaT sensor cells provide a means for the automated detection of the compromised redox status of keratinocytes as an early indicator of the development of human skin disorders and could be applied for the prediction of skin irritation in more complex *in vitro* 3D human skin models and in the development of micro-total analysis systems (μTAS) that may be utilized in dermatology, toxicology, pharmacology and drug screenings.

## 1. Introduction

Allergic and irritant contact dermatitis are the most common eczematous diseases. An early response of keratinocytes, which constitute the major epidermal cells of the skin, involves a non-immunological reaction that is characterized by cell damage induced by reactive oxygen species (ROS). ROS can promote the release of pro-inflammatory cytokines from keratinocytes, which in turn activate an immunological response that leads to the progress of contact dermatitis [1,2].

There are several sources of ROS in the cell, the most notable resulting from alterations in the activities of NADPH oxidase, xanthine oxidase and the mitochondrial respiratory cycle. If chemicals interfere with the activities of these enzyme systems, oxidative stress will be rapidly elevated. Evaluation of the cell-damaging effects of compounds including oxidative stress can be conducted by cell-based sensor systems, which are capable of adapting the process of skin irritation by integrated *in vitro* models and enabling a predictive as well as descriptive prescreening. Moreover, they may represent a sufficient alternative to the three common ways of measuring the presence of oxidative stress, which are the direct measurement of ROS, the determination of the ROS-evoked damage to biomolecules, and the detection of antioxidant levels. Their spectra have been intensively reviewed [3]. Direct detection is hampered by its extreme instability of ROS. Therefore, it is preferred to measure the ROS-induced damage to proteins, DNA, RNA, lipids, or other biomolecules by adapting thiobarbituric acid (TBA) or Comet assays, among others. While these are indirect methods, many markers of damage are extremely stable and therefore provide a more reliable scheme of measuring oxidative stress. Another approach is to quantify the levels of antioxidant enzymes and other redox molecules that serve to counterbalance ROS generated in the cell. Assays are available to measure the activity of specific antioxidant enzymes, such as catalase and superoxide dismutase, or the determination of redox-sensitive systems such as thioredoxin or glutathione [4]. The latter exists in two forms, including the reduced sulfhydryl form (GSH) and the oxidized disulfide (GSSG). The main function of GSH is to neutralize ROS using mechanisms involving glutathione peroxidase, glutathione-S-transferase and glutathione reductase. Interestingly, glutathione concentrations are significantly increased in HaCaT cells following exposure to the toxic metal cadmium [5,6]. Although the mechanisms of cadmium toxicity are cell type-dependent, this metal is known to induce the expression of cysteine-rich, metal-binding proteins (metallothioneins) that can protect the cell from cadmium toxicity and inactivate antioxidant enzymes by interacting with the thiol groups of these proteins, resulting in the generation of ROS. In addition, cadmium provokes homeostatic alterations in physiological metals, such as copper, zinc and iron, and increases the transcript levels of enzymes that are involved in glutathione biosynthesis, including gamma-glutamylcysteine synthetase and glutathione synthetase. Cadmium severely affects the redox status of cells, even at sub-lethal concentrations and induces oxidative stress in HaCaT cells as demonstrated by decreased GSH/GSSG ratios [5–7].

The human skin is permanently exposed to stress factors, such as ultraviolet light and xenobiotics, which may result in the generation of ROS. As such, keratinocytes are actively involved in the immune reactions that are characteristic of contact dermatitis, for example, by producing cytokines. Therefore, keratinocytes represent a valuable model cell type for the screening of the dermatological effects of skin-damaging agents. In this study, a simple and fast detection of the oxidative stress response in keratinocytes is described using genetically modified sensor cells that are based on the Human adult low Calcium high Temperature (HaCaT) cell line [8], which was stably transfected with a reporter construct that allowed for the expression of green fluorescent protein (GFP) under the control of the promoter regulating the human heat shock protein HSP70B'. The resulting optical sensor cell line demonstrated time- and dose-dependent activities when chemicals or plant extracts that are known skin irritants were used. We compare our results to conventional measurements of total glutathione (tGSH) concentrations in HaCaT cells and highlight the advantages of using a live cell-based biosensor instead. Furthermore, we discuss insights into the varying effects of xenobiotics on non-immunological skin cells, thereby contributing to the understanding of keratinocyte participation in the development of different forms of contact dermatitis.

Based on our recent study that established HSP72 as a biomarker for the indirect determination of oxidative stress in cultured human keratinocytes [9], we evaluated here whether the properties of this keratinocyte sensor cell line could be improved for future applications in micro-total analysis systems (μTAS) by replacing the HSP72 promoter with that of HSP70B'.

## 2. Experimental Section

### 2.1. Cell Culturing and Cytotoxicity Testing

The cultivation of HaCaT cells [8] and determination of cell viability using the 3-(4,5-dimethylthiazol-2-yl)-2,5-diphenyltetrazolium bromide (MTT) cytotoxicity assay were performed as described previously [9]. Percent cytotoxicity was calculated by dividing the absorbance of the treated cells by that of the untreated controls. The stock solution of 2,4-dinitrochlorobenzene (DNCB) was prepared in ethanol, nickel sulfate (NiSO_4_·6H_2_O) and cadmium chloride (CdCl_2_) were dissolved in deionized distilled water.

### 2.2. Determination of Gene Expression Levels

The isolation of total RNA from the HaCaT cells and quantitative reverse transcription-polymerase chain reaction (qRT-PCR) were essentially performed as described [9]. The primer sequences for qRT-PCR were as follows: HSPA6 fwd 5′-TGC AAG AGG AAA GCC TTA GGG ACA-3′ and rev 5′-TTT GCT CCA GCT CCC TCT TCT GAT-3′, GAPDH fwd 5′-TTC GAC AGT CAG CCG CAT CTT CTT-3′ and rev 5′-GCC CAA TAC GAC CAA ATC CGT TGA-3′. All qRT-PCR measurements were conducted at least three times from three independent cell cultures. The data were analyzed using Student's *t*-test.

### 2.3. Arnica Montana Extract

Dried flowers from the *A. montana* L. plant were extracted by PHARMAPLANT GmbH (Artern, Germany) in 70% ethanol (v/v) at 60 °C for 36 h. The solvent was evaporated to dryness after first being reduced using a vacuum rotary evaporator at 60 °C. For the cell culture experiments, the dry extracts (drug-extract ratio of 7.3:1) were dissolved in dimethyl sulfoxide. For the quantitative analysis, sample preparations were conducted according to the previously described method [10] with an amount of extract that was equivalent to 1 g of dried plant material using santonin as an internal standard. The analysis was conducted using the Agilent GC/MS system (GC 7890A, MSD 5975C, Gerstel GmbH & Co. KG, Mühlheim an der Ruhr, Germany) that was equipped with a SLB™-5 ms chromatographic column with 60 m × 0.25 mm I.D. × 0.25 μm film thickness (Sigma-Aldrich, Taufkirchen, Germany). The initial column temperature was 100 °C, which then increased from 100 °C to 300 °C at 10 °C/min and held at 300 °C for 40 min. The injector temperature was 250 °C and the injection volume was 1 μL. The carrier gas flow was 1.6 mL/min with a 1:10 splitting ratio.

### 2.4. Engineering the Sensor Cell Line

A genomic region corresponding to nucleotides −641 to +110 of the *HSPA6* gene (+1 referring to the transcription start) was amplified from the human genome by PCR using 5′-AAA AAC TCG AGA CCA CTG AAC CAC CAA TGC T-3′ as the forward primer and 5′-AAA AAC CGG TCT TCT TGT CGG ATG CTG GA-3′ as the reverse primer. The resulting DNA fragment containing the functional promoter region of *HSPA6* [11] was subcloned into the XhoI and AgeI sites of pAcGFP1-1 (Clontech, Saint-Germain-en-Laye, France) to generate pHSP70B'_p_-AcGFP1-1. The stable transfection of the HaCaT cells was achieved using TurboFect (Thermo Fisher Scientific, Schwerte, Germany) according to the manufacturer's protocol and a subsequent selection in medium containing 1 mg/ml G418. Resistant cells were harvested and initially screened using fluorescence-activated cell sorting (FACS) (FACSAria IIU, Becton Dickinson, Heidelberg, Germany) to eliminate cells with constitutively high GFP expression levels and no induction of the functional promoter (negative screening). Cells demonstrating low basal GFP expression were tested for HSP70B'-induced GFP expression by incubation for 2 h at 43 °C followed by a 12–16 h recovery at 37 °C. Only cells responding to heat shock with high levels of GFP expression were collected for single clone selection (positive screening). HSP70B'-induced GFP expression following heat shock was confirmed in single clones by microscopy and FACS to select a working cell line showing the strongest signal-to-noise ratio in response to heat shock (2 h, 43 °C) and exposure to CdCl_2_ (6 h, 25 μM) [9].

### 2.5. Quantitative Fluorescence Microscopy

Fluorescence microscopy was used for the quantitative assessment of cellular GFP expression levels in an incubation time- and dose-dependent manner. The sensor cell line was seeded on coverslips at a density of 50,000 cells/cm^2^ in 24-well culture plates. Following treatment with toxins, the cells were kept under normal culture conditions for up to 18 h. GFP expression levels were observed using an Axio Imager M2 (Zeiss, Jena, Germany). Cell culture images showing confluent cell layers were digitally recorded with an AxioCam MRm camera that was attached to the microscope using the filter set 38 HE and a 20× objective (Zeiss, Jena, Germany). The average fluorescence (mean grey value) of each cell region was corrected for medium and device fluorescence, and compared to an untreated control to obtain relative fluorescence values.

### 2.6. Determination of Total Cellular Glutathione

Concentrations of tGSH in cells were determined as described previously [12]. Two hundred thousand cells were seeded into each well of a 6-well plate. Following incubation with the toxins, the cells were washed with PBS. After harvesting, 600,000 cells were resuspended in extraction buffer (0.1% Triton X-100 and 0.6% 5-sulfosalicylic acid in potassium phosphate buffer) and underwent two cycles of freezing at −80 °C and thawing. The lysates were centrifuged, and the supernatants were mixed with 5,5′-dithio-bis-(2-nitrobenzoic acid (DNTB, 0.66 mg/mL), glutathione reductase (454 U/mL) and NADPH (0.66 mg/mL). The chromophore product was immediately measured at 412 nm every 30 s for 3 min. The tGSH concentration of the unknown sample was determined according to a regression curve of the GSH standard.

## 3. Results and Discussions

### 3.1. HSP70B' is a Sensitive Biomarker for the Prediction of Oxidative Stress

HSP70B' was previously used as a biomarker for oxidative stress in diverse mammalian cell lines [13–15]. Building upon these observations, we first tested whether HSP70B' expression was also induced in response to stress in the human keratinocyte cell line HaCaT. To this end, HaCaT cells were exposed to either 25 μM CdCl_2_ or elevated culture temperatures (43 °C), which are both known to induce HSP72 promoter activity in HaCaT cells [9]. The method of qRT-PCR was used to determine the mRNA levels of the HSP70B' gene. We found that its expression was low in untreated cells and was induced by approximately 335-fold in response to CdCl_2_ exposure (data not shown). Likewise, 148-fold and 269-fold inductions in HSP70B' gene expression were observed following heat treatment for 1 h and 2 h, respectively. The observation of a stronger induction from very low basal activity compared to HSP72 indicated that the generation of a sensitive sensor cell line based on a HSP70B'-driven reporter gene in HaCaT cells would be superior to the HSP72-GFP reporter in future μTAS settings.

**Figure 1.**
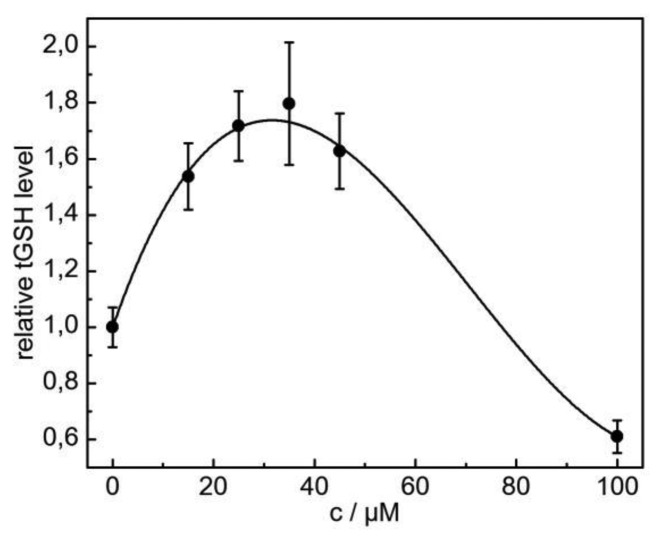
Glutathione pool in HaCaT cells increases following exposure to CdCl_2_. Total GSH levels increase in HaCaT cells following exposure to 15, 25, 35, 45 and 100 μM CdCl_2_ for 6 h [mean ± SE; n = 3]. The curve was fitted to the experimental data using a polynomial regression analysis.

To determine whether the induction of HSP70B′ expression in HaCaT cells in response to CdCl_2_ exposure was in fact correlated with increased oxidative stress, we determined tGSH concentrations in transfected cells. Oxidative stress can act in three different ways on the glutathione pool in HaCaT cells [6]. First, CdCl_2_ may alter the ratio of the reduced sulfhydryl form of glutathione (GSH) to the oxidized glutathione disulfide (GSSG). Second, CdCl_2_ exposure affects the activities of enzymes that are involved in combatting ROS, such as superoxide dismutase, catalase, and glutathione peroxidase. Third, treatment with CdCl_2_ increases tGSH concentrations in the cells. As shown in Figure 1, a dose-dependent increase in tGSH concentrations in HaCaT cells in response to a CdCl_2_ challenge was observed. The maximal increase in tGSH of approximately 1.7-fold was achieved following the treatment of cells with 25–45 μM CdCl_2_ for 6 h, which was well below the range of general CdCl_2_ cytotoxicity in HaCaT cells as determined by MTT assays following 24 h of exposure (EC_50_ = 45 ± 7 μM) [9]. The limit of detection (LOD) of the applied tGSH assay was determined to be 1 μM CdCl_2_ following a 6 h incubation with the toxin. A CdCl_2_-induced increase in tGSH could be explained in principle by the oxidative stress-induced activation of signaling pathways that led to an increase in the *de novo* production of enzymes that are involved in glutathione biosynthesis [16,17].

**Figure 2.**
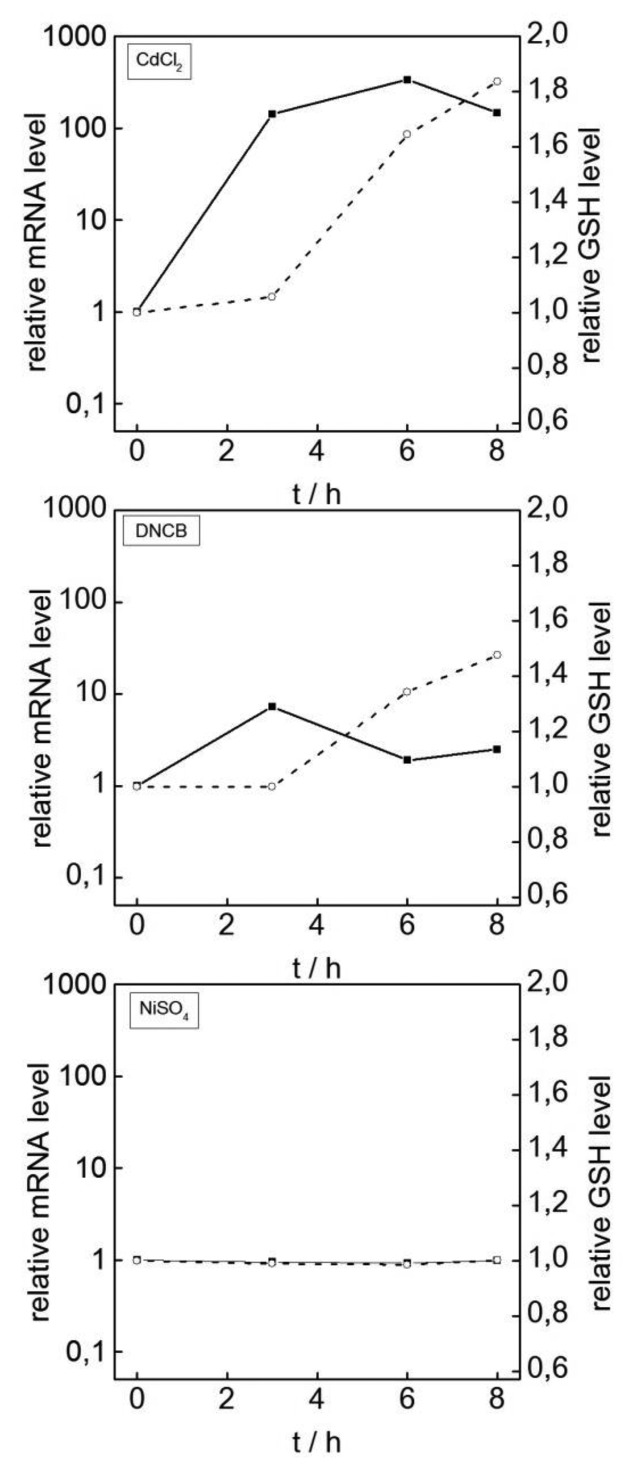
Responses of HaCaT cells to treatments with DNCB and NiSO_4_. HSP70B' expression (detected by qRT-PCR) and changes in tGSH levels were measured following the incubation of HaCaT cells with 25 μM CdCl_2_, 10 μM DNCB, or 400 μM NiSO_4_. Solid lines represent the detection of the relative mRNA levels of HSP70B' compared with untreated cells. Dashed lines represent the relative tGSH levels. For each panel, average values from 3–6 independent experiments are shown.

To further evaluate the suitability of HSP70B' induction as a predictor of oxidative stress-induced cell damage in keratinocytes, HSP70B' gene expression was tested following the exposure of HaCaT cells to selected chemicals and a plant extract that was prepared from *A. montana* flowers, which are known skin irritants [18]. Concentrations of compounds were selected according to their general cytotoxicities to HaCaT cells according to EC_80_ values as determined by MTT assays after 24 h of exposure (data not shown). We first determined in parallel the stress-induced increase in HSP70B' expression and the cellular tGSH content after challenging the cells with 10 μM of DNCB or 400 μM of NiSO_4_ for up to 8 h. The response of cells to 25 μM CdCl_2_ served as a positive control (Figure 2). The experiments reproduced the 335-fold induction of HSP70B' expression following CdCl_2_ treatment that persisted over an 8-hour period. Likewise, a nearly 2-fold increase in tGSH content was achieved in cells that were treated with CdCl_2_ (Figure 2). An approximately 7.3-fold induction in HSP70B' expression was observed following the exposure of cells to DNCB for 3 h. This response was not only much less pronounced compared with the cadmium-induced effects, but it also rapidly reverted to nearly normal levels after 6–8 h. Nevertheless, DNCB induced an approximately 1.5-fold increase in the cellular tGSH pool. Interestingly, the treatment of cells with 400 μM of NiSO_4_ neither induced HSP70B' expression nor did it enhance the cellular tGSH pool (Figure 2). This is consistent with the observation that NiSO_4_ does not produce ROS in a concentration range that is non-toxic to HaCaT cells [2]. In summary, the data show that HSP70B' is a suitable biomarker for chemicals that induce oxidative stress irrespective of whether they act as a skin irritant (CdCl_2_) or cause allergic contact dermatitis (DNCB) *in vivo*

**Figure 3.**
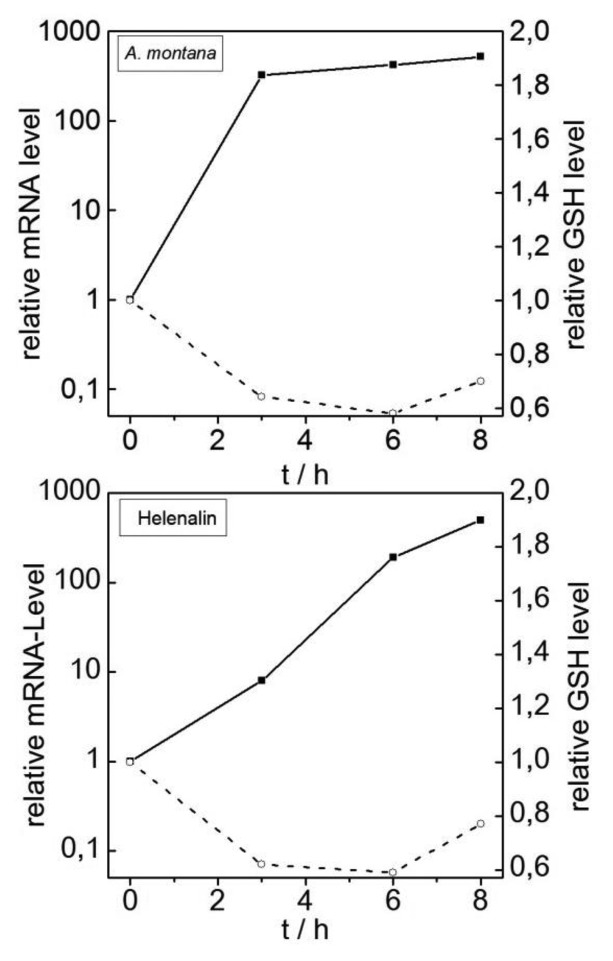
Responses of HaCaT cells to treatments with *Arnica montana* extract and helenalin. HSP70B' expression (detected by qRT-PCR) and changes in tGSH levels were measured following the incubation of HaCaT cells with 125 μg/mL *A. montana* or 5 μM helenalin. Solid lines represent the detection of the relative mRNA levels of HSP70B' compared with untreated cells. Dashed lines represent the relative tGSH levels. For each panel, average values from 3–6 independent experiments are shown.

Dried flowers of *A. montana* are used for the topical treatment of skin bruises, contusions, and pain. Despite its traditional anti-inflammatory use [19], there have been several reports attributing dose-dependent cytotoxic effects to the sesquiterpene lactone (STL) fraction of the *A. montana* extract, particularly helenalin [18]. STL can cause contact dermatitis in two principal ways; it can directly alkylate and inactivate proteins that are involved in the cell-protective stress response. Given the millimolar concentration of glutathione in the cell, STL rapidly form glutathione adducts, which can also act as specific inhibitors of enzymes that are required, for example, to combat oxidative stress [20,21]. We investigated the impact of the *A. montana* extract and purified helenalin on HSP70B' expression in HaCaT cells. The qRT-PCR measurements revealed a strong dose- and time-dependent increase in HSP70B' mRNA levels after the treatment of HaCaT cells with the *A. montana* extract. Treating the cells with the EC_80_ concentration of the *A. montana* extract (125 μg/mL) resulted in a >300-fold induction in HSP70B' gene expression after 3 h of incubation (Figure 3). A quantitative analysis of the *A. montana* extract that was used in this study revealed a concentration of 144.0 ± 26.7 μg/mL or 530 μM of helenalin. Considering that we applied the extract according to the EC_80_ value of 125 μg/mL, the final concentration of helenalin in the experiment was estimated to be 1.35 μM. Pure helenalin was able to increase HSP70B' mRNA expression in a dose-dependent manner with saturation at ∼7 μM (data not shown). If applied at its EC_80_ concentration of 5 μM, helenalin induced HSP70B' mRNA expression to similar levels as the *A. montana* extract (Figure 3). The induction of HSP70B' expression by helenalin is consistent with its reported stimulation of apoptosis by the generation of ROS [22]. The results suggest that helenalin is a major active component in the *A. montana* extract with respect to the observed HSP70B' induction, but further components of the STL or flavonoid fractions may have additive or synergistic roles in *A. montana* cytotoxicity in HaCaT cells.

It is worth noting that although its effect on HSP70B' expression was similar to that which was observed following CdCl_2_ treatment, the tGSH concentrations of cells that were exposed to the *A. montana* extract did not increase but rather declined by more than half of the level of untreated controls (Figure 3). Likewise, helenalin dramatically reduced the tGSH pool in the HaCaT cells. Although the effects on the cellular tGSH concentrations that were obtained using the *A. montana* extract or helenalin seemed to conflict with the results that were observed following exposure to cadmium, other studies have described the reduction of the cellular tGSH pool by STL, including helenalin, likely through the ROS-dependent oxidization of the cysteine pool that is required for glutathione synthesis [23].

The molecular mechanisms underlying the differential effects of CdCl_2_ and helenalin on the cellular tGSH pool remain elusive; however, our observations may indicate that different gene regulatory pathways are activated by either compound, irrespective of the fact that both are strong inducers of ROS production. Whereas the toxic mechanisms of cadmium have been thoroughly described [6,7,24], the effects of contact allergens, such as DNCB and NiSO_4_, on non-immunological skin cells have been investigated only recently [2]. Although concentrations of compounds were applied that had comparable effects on cell viability (EC_80_), the incubation of HaCaT cells with CdCl_2_ resulted in a much higher induction of HSP70B' compared with the DNCB treatment. The toxic mechanisms of cadmium that affect cellular homeostasis are very complex and act indirectly by inhibiting antioxidant enzyme activities, leading to increased ROS levels [5,25]. Furthermore, the heat shock factor HSF-1 has been identified as the mediator of stress-induced heat shock gene expression on the basis of its ability to display inducible DNA binding activity, oligomerization, and nuclear localization in response to environmental stressors, such as elevated temperatures and cadmium [26]. These multilevel toxic mechanisms may explain the somewhat stronger signal that is substantiated by the incubation time-dependent increase and saturation of the mRNA expression levels as determined by qRT-PCR and the increased tGSH levels. The general instability of DNCB combined with the short-term ROS-buffering capacity of the GSH/GSSG pool may affect the redox balance of HaCaT cells much less than cadmium-induced damage.

In summary, HSP70B' is a well-suited biomarker for the detection of a deregulated oxidative stress level by diverse chemical compounds as an increased HSP70B' expression correlates with tremendous changes of the intracellular tGSH pool independent of the underlying specific toxic mechanisms. Thus, we next developed a sensor cell line based on the HSP70B' promoter for the rapid and sensitive *in vitro* screening of chemicals that are suspected to cause skin irritation.

### 3.2. Design of an Oxidative Stress Sensor Cell Line in HaCaT Cells

HaCaT cells were stably transfected with a plasmid that allows for the expression of GFP under the control of the HSP70B' promoter. To establish the reporter gene system, we first tested for the induction of GFP fluorescence in different clones of stably transfected cells after the exposure of the sensor cells to CdCl_2_ and heat shock. For each clone of sensor cells, the absolute emission output and dynamic range of GFP induction in stress situations was evaluated using flow cytometry (data not shown). Based on these results, a single clone that showed the best signal-to-noise ratio and highest dynamic range following treatment for short time periods was selected for further investigations. The threshold point was determined to be the fraction including approximately 95% of control cells that were not exposed to stress (intrinsic fluorescence). Then, the number of GFP-positive cells was calculated. The fraction of GFP-positive cells following exposure of the sensor cells to 25 μM CdCl_2_ for 6 h was 52.5%, and 49.7% of the cells were GFP-positive after 2 h of exposure to 43 °C.

**Figure 4.**
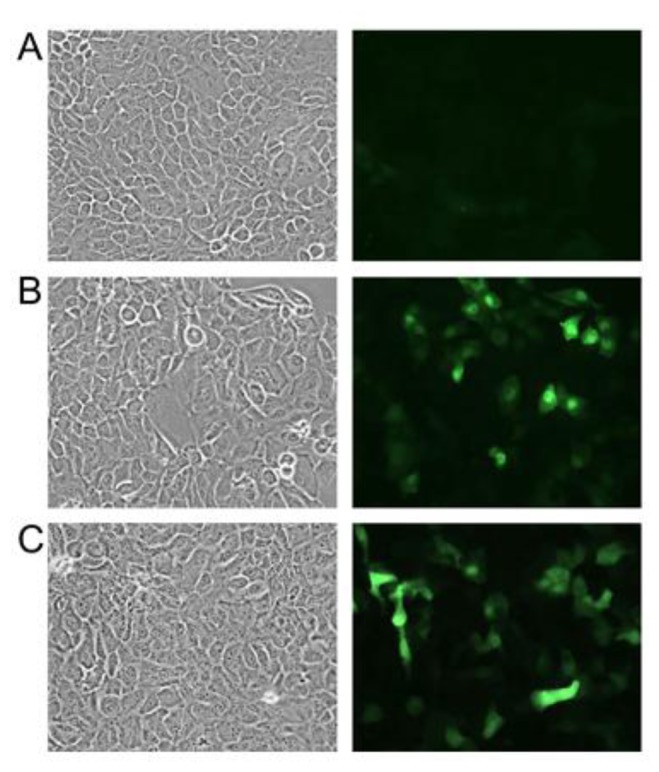
Fluorescence microscopy of HSP70B'-GFP sensor cells. Phase contrast images (left panels) and GFP fluorescence (right panels) of either untreated cells (**A**) or cells that were treated for 6 h with 25 μM CdCl_2_ (**B**) or 5 μM helenalin (**C**).

In the next series of experiments, the cells were seeded on coverslips for fluorescence microscopy to quantitatively investigate the expression levels of GFP in an incubation time- and dose-dependent manner. The cells were grown to near confluence and then treated with chemicals to induce oxidative stress. The results are presented in Figure 4 for the 25 μM CdCl_2_ and 5 μM helenalin treatments. The microscopic images indicated a low basal fluorescence of the sensor cells that considerably increased in response to stress.

**Figure 5.**
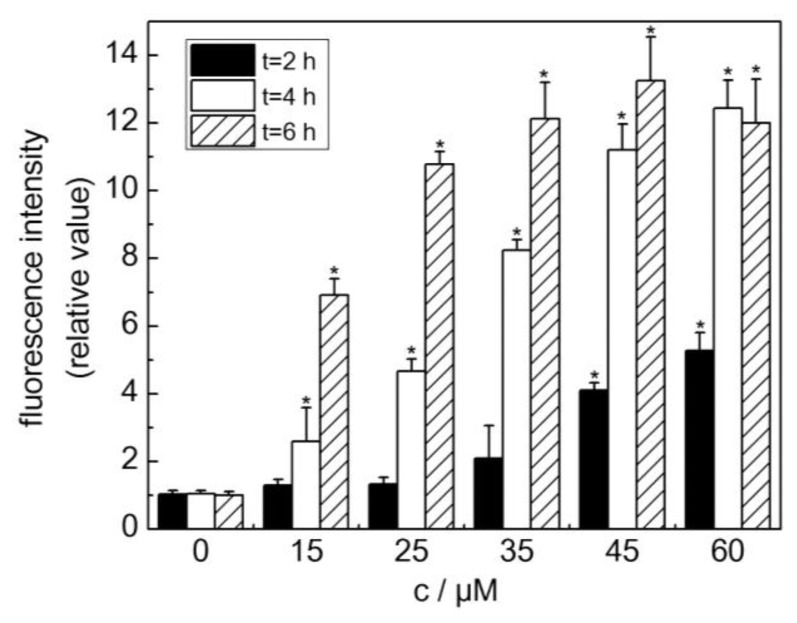
Quantification of GFP fluorescence following exposure of sensor cells to CdCl_2_. The responses of sensor cells to treatments with 15, 25, 35, 45 and 60 μM CdCl_2_ for 2, 4, and 6 h were analyzed by quantitative fluorescence microscopy. Relative fluorescence intensity relates to the corresponding values for samples from the untreated cells, which was set as 1 [mean ± SE; n = 3; *P < 0.05].

A quantitative read-out of the stress response that was elicited by the HSP70B'-GFP cells was desirable for the facilitation of the automated screening of the chemicals that were suspected to cause skin irritation. To this end, oxidative stress-induced GFP fluorescence was determined using quantitative fluorescence microscopy. As presented in Figure 5, the exposure of the sensor cells to increasing CdCl_2_ concentrations induced a concentration- and time-dependent increase in GFP fluorescence that reached a plateau at approximately 12-fold levels of induction compared with the untreated sensor cells. The exposure of the sensor cells to over 60 μM CdCl_2_ resulted in aberrant cell morphology and eventually decreased GFP fluorescence (data not shown). Fluorescence intensities were recorded for different CdCl_2_ concentrations after 6 h of incubation to determine the lowest detectable concentration. The LOD was defined as the concentration that induced a fluorescence intensity that was equal to or higher than the mean fluorescence of the untreated control cells plus three times the standard error of the measurement according to the three-sigma rule (data not shown). Based on this definition, the sensor cells displayed an LOD of 6 μM CdCl_2_ for a 6 h exposure time, which is comparable to the LOD of the applied conventional tGSH assay.

When the cells were treated with either DNCB, the *A. montana* extract or helenalin, all of which produce oxidative stress, similar levels of fluorescence induction were recorded (Table 1). It is worth noting that the treatment of sensor cells with NiSO_4_ elicited no response, which is in accordance with the aforementioned data indicating that NiSO_4_ does not cause oxidative stress in HaCaT cells. The data has also been compared with the values achieved by the former sensor cell line based on HSP72. The novel sensor cell line exhibits a comparable response profile but represents a significant improvement due to its 1.5-fold increased signal-to-noise-ratio. These data suggest that the HSP70B'-based sensor cells are suitable for the quantitative detection of ROS-induced cytotoxicity under sublethal conditions and shorter incubation periods compared with the endpoint measurements.

Compounds were applied at their EC_80_ concentrations of general cytotoxicity as determined by the MTT assay. Relative GFP fluorescence was standardized to the untreated cells. For each data set, mean values were calculated from 3–6 independent experiments [± SE].

## 4. Conclusions

In this study, we used genetically modified sensor cells expressing a HSP70B′-GFP reporter construct to demonstrate that it is possible to sensitively and rapidly quantify an early skin irritation response upon exposure to a panel of test substances that are known to cause irritant or allergic contact dermatitis. Furthermore, these data support the view that varying effects of xenobiotics impact non-immunological skin cells, supporting the importance of further research on keratinocyte participation in the development of pathologic cell states. Independent of the signaling pathways that are employed or the effects that chemicals or even complex mixtures of natural products may exhibit to cause oxidative stress, it was shown here that HSP70B' expression and the derived reporter gene output serve as a valuable detection setup that enabled the construction of a rapid and sensitive *in vitro* pre-screening test for oxidative stress dysregulation, which is a fundamental characteristic that appears early in the onset of human skin disorders.

We compared the performance of the novel HSP70B'-based assay to conventional measurements of tGSH concentrations and received similar response profiles as well as LODs following 6 h of incubation (tGSH: 1 μM CdCl_2_ and whole-cell biosensor: 6 μM CdCl_2_). The sensory method is characterized by a rapid absolute workload due to the elimination of sample preparation; it is non-destructive, and, consequently, adaptable to real-time measurements as well as other non-invasive detection procedures such as impedance readouts.

Compared with the previously established HSP72-GFP reporter cells [9], the sensor cell line that was described here represents a significant improvement due to its superior signal-to-noise-ratio that is required for optimal sensor integration towards the development and evaluation μTAS for application in dermatology, toxicology, pharmacology and drug screenings. The integration of the developed toxicity assay into a μTAS has the big advantage that it works as shown in this paper under incubator-free conditions. The constant microfluidic stream of culture medium together with the temperature control und the use of CO_2_-independent medium for stabilized pH enables the creation of microsystems incorporating several steps of an assay into a single system. Under such conditions miniaturized complex systems for a broad field of applications can be easily developed. Such integrated microfluidic devices perform rapid and reproducible measurements on small sample volumes while eliminating the need for expensive instrumentation and labour-intensive laboratory manipulations.

## Figures and Tables

**Table 1.** Response of HSP72-GFP [9] and HSP70B′-GFP sensor cell to different forms of stress.

Substance	Compound (EC_80_)	Response HSP72-GFP (Relative Fluorescence)	Response HSP70B' (Relative Fluorescence)
	t = 24 h	t = 6 h	t = 6 h
CdCl_2_	25 μM	7.7 ± 0.3	10.4 ± 0.3
DNCB	10 μM	4.7 ± 0.7	6.9 ± 0.2
NiSO_4_	400 μM	1.2 ± 0.2	1.1 ± 0.1
*A. montana*	125 μg/mL	5.5 ± 0.3	8.1 ± 0.2
Helenalin	5 μM	not tested	9.8 ± 0.3
